# Overcoming barriers to the involvement of deafblind people in conversations about research: recommendations from individuals with Usher syndrome

**DOI:** 10.1186/s40900-018-0124-0

**Published:** 2018-10-26

**Authors:** Andrew Skilton, Emma Boswell, Kevin Prince, Priya Francome-Wood, Mariya Moosajee

**Affiliations:** 1 UCL Institute of Ophthalmology, 11-43 Bath Street, London, EC1V 9EL UK; 2 Moorfields Eye Hospital NHS Foundation Trust, 162 City Road, London, EC1V 2PD UK; 3 Sense, 101 Pentonville Road, London, N1 9LG UK; 4 Research Community Contributor, NIHR Moorfields Biomedical Research Centre, 162 City Road, London, EC1V 2PD UK; 5 Great Ormond Street Hospital for Children NHS Foundation Trust, Great Ormond Street, London, WC1N 3JH UK

**Keywords:** Usher syndrome, Deafblind, Deafblindness, Accessibility, Sight impairment, Hearing impairment, Involvement, Participation, Research, Communication

## Abstract

**Plain English summary:**

Usher syndrome is the most common cause of deafblindness worldwide and is estimated to affect between 3 and 6 people in every 100,000. Children are born with hearing loss and develop sight loss in their early years of life. A barrier to the involvement and participation of deafblind people in research is access to information in appropriate formats. The degree of sight and hearing impairment experienced by individuals is variable, so there is not a one size fits all solution. We held a research discussion group, that included five people with Usher syndrome, to consider people’s accessibility needs for an upcoming research project involving this condition.

We have identified a number of considerations for including deafblind people in conversations about research: i) using appropriately sized meeting rooms which offer control over lighting, layout and sound; ii) where appropriate, ensuring written/printed materials are high contrast (e.g. black text with a yellow background) and in large (18 point and above), sans-serif fonts (e.g. Arial); iii) identifying the relevant communication support for the individual whether that be sign language interpretation, lip reading, hearing loop, speech to text reporting or a combination; iv) ensuring that there is access to emotional support for both people who are deafblind and their families before, during and after the research.

The outcome of this work is a checklist of considerations when planning to hold a research conversation with someone who is deafblind and hinges on earlier interactions to identify the appropriate support needs for the individual.

**Abstract:**

**Background**

Usher syndrome is the most common cause of deafblindness worldwide. Children are born with hearing loss and develop sight loss in their early years of life. It is estimated to affect between 3 and 6 people in every 100,000. A barrier to the involvement and participation of deafblind people in research is access to information in appropriate formats. Individuals have varying degrees of sight and hearing impairment meaning there is not a singular solution to supporting all people’s communication needs. There is evidence that severe sight and hearing impairments are used as exclusion criteria in some research studies. This exclusion may extend into involvement activities.

**Methods**

Eight people, including five people with Usher syndrome, attended a research discussion group. Through this activity, we identified what to consider when looking to improve the experience of taking part in a discussion about research for deafblind individuals.

**Results**

Among contributors two people made use of standard British Sign Language interpretation and one communicated using hands-on signing. Contributors highlighted the limitations associated with signing and lip reading such as exhaustion and clear lines of sight as well as the need for additional formats such as speech to text reporting, and high contrast (e.g. black text with a yellow background) printouts with large (18 point and above), sans-serif fonts (e.g. Arial). A large proportion of discussions were on the importance of wrap around emotional support for people who are deafblind and their family throughout the research pathway. This includes counselling, peer support and sensitive and mindful facilitators of involvement activities.

**Conclusions**

The range and specific nature of the communication methods and support offerings that deafblind people depend on are broad and require researchers and involvement practitioners to reach out to deafblind contributors earlier on, in order to appropriately tailor approaches and put the most suitable support in place. Informed by this discussion group, we have developed a checklist of key considerations to support the inclusion of deafblind individuals in research conversations, supplemented with input from the sensory disability charity Sense.

## Background

A barrier to deafblind people being involved in research (the inclusion of members of the public as decision makers in research) or participating in research (the enrolment of people into research) is access to information in accessible formats. While there are various sources of advice and guidance on communicating with this audience [1, 2], these can be difficult to find if you are unsure where to look. Previous reports on the health literacy and mental well-being of people with sight and hearing impairment may also raise concerns over the ability of this group to provide informed consent for clinical study enrolment [3–8]. Unfairly, deafblind people may be seen as a group that is too difficult to include in conversations about health research [3, 5].

There is evidence that in the past sight and/or hearing impairments have been used to exclude potential participants from taking up opportunities to enrol in research [9–11]. The PaRticipation of the ElDerly In Clinical Trials (PREDICT) study sought to establish supporting evidence and develop ethical standards for the inclusion of older people in clinical trials [9, 11]. A sub-analysis of 251 heart failure studies found that 11 studies excluded those who had sight or hearing loss from participating, and that nine of these exclusions were ‘poorly justified’ and not made on the grounds of safety, i.e. there was no risk of further sight or hearing loss as a direct result of participating [9]. More recently, the Improve Cardiovascular Outcomes in high-risk patieNts with acute coronary syndrome (ICON-1) longitudinal study published on the feasibility of recruiting older people. While exclusion criteria for ICON-1 did not include sight or hearing loss, ‘severe’ sight or hearing impairments were listed as the reason potential participants were unable to give informed consent in nine cases [10].

PREDICT has gone on to develop a “charter for the rights of older people in clinical trials”, which advocates for reasonable support, researcher training and adaptions to the informed consent process to support the participation of those with sight and hearing impairment [9]. What is not clear, however, is to what extent these sensory impairments alone were reasons to exclude potential participants from the studies identified in PREDICT, and whether the charter was considered before decisions not to include this group were made for ICON-1.

While overall reports of sight or hearing impairment as an exclusion criterion for research in the literature are few, this is most likely due to underreporting. The studies mentioned here raise the question; to what extent do such exclusions, and a lack of planning for adequate adaptions in research design for those with more complex dual sensory impairment, impact negatively on the deafblind?

Usher syndrome (USH) is a genetic condition characterised by hearing loss from birth (resulting from faults in the structure and function of the inner ear canal) and progressive sight loss caused by degeneration of the retina, the light-sensing layer at the back of the eye [12]. Worldwide, it is the most common cause of deafblindness, estimated to affect between 3 and 6 people in every 100,000 [12]. This condition is lifelong and there are no treatments available. For people with USH, progressive visual and hearing impairments occur in early childhood with many individuals experiencing severe deafblindness before 40 years of age [5, 12].

As of October 2018, there were four clinical trials involving people with USH registered on clinicaltrials.gov and the World Health Organisation’s International Clinical Trials Registry Platform [13, 14]. The existence of these studies demonstrate that having sensory impairment does not preclude one from being able to provide informed consent to participate in a study. The process of informed consent involves discussion of the intervention under investigation, the research protocol and the risks versus benefits of the study. These same topics often feature in conversations as part of involvement activities. Therefore, this would infer that people with sensory impairments are able to take part in a meaningful conversation about research in an involvement context as well.

There may be a number of perceived challenges to communicating with deafblind individuals that those less familiar with the condition might be concerned with trying to overcome. People who are deafblind often have varying degrees of visual and hearing impairment, which means there is not a singular solution to meeting peoples accessibility requirements [3, 12]. While it is relatively simple to produce written materials in more visually accessible formats [15, 16], there are more significant barriers to supporting those with severe hearing loss; namely the costs associated with providing access to sign language interpreters with further speciality skills such as hands-on deafblind manual signing and experience with signing for a health-research context [3, 4]. Anxiety about communication barriers, obstacles such as limited budgets and a lack of training in accessibility and sensory impairment awareness may aid in labelling individuals with USH, and those with other forms of deafblindness, as a ‘hard-to-reach’ audience [3, 4].

Here, we report on our learnings from a research discussion group undertaken in 2017 which included people with USH with varying degrees of sight and hearing impairment. From observations during the discussion group, input from those with USH who took part, as well as additional input from the sensory disability charity Sense, we have collated recommendations for the inclusion of deafblind people in conversations about research along with a checklist of key considerations to support others in planning such activities.

## Methods

### Procedure

Eight people attended a 4-h research discussion group, with 30-min breaks between sessions. Five of these contributors had USH with varying degrees of sight and hearing loss between them. Contributors were recruited from the hospital electronic patient records or via the charity Sense. All contributors were unpaid volunteers.

One USH contributor had a guide dog and another used a hearing aid. Two USH contributors required British Sign Language (BSL) interpretation and one needed hands-on deafblind manual signing (physically signing on the deafblind person’s hands) to take part in discussions. Contributors provided their own interpreter support. There were three interpreters present at the session; none were active contributors to conversations. None of our other contributors required any additional communications support to take part.

Discussions were held outside of the hospital setting in a meeting room at UCL Institute of Ophthalmology and facilitated by NIHR Moorfields Biomedical Research Centre’s patient and public involvement (PPI) lead (AS). The principal investigator (MM), the discussion group organiser who is a genetic counsellor (PF) and a fourth colleague from the research team were also in attendance. In addition to the availability of light refreshments and sandwich lunch, contributors were able to claim up to £30 to reimburse their travel expenses.

### Collating recommendations

We report on how we met the accessibility needs of deafblind people in research involvement as well as where we would need to improve for the future. Two contributors with USH from the discussion group are co-authors on this paper, KP and EB, who is the National Usher Co-ordinator for the charity Sense. KP and EB provided further recommendations to make the findings of this paper as broadly applicable as possible. Direct quotes from contributors (C1, C2, C3, C4), are taken from audio recordings of the discussion group made with the consent of the contributors.

## Results

The opinions and experience-sharing that arises from conversations about research with contributors with lived experience of a condition are valuable to ensure research design is appropriate for those who participate [17–20]. There are also insights to be drawn from independent observations of how individuals interact within a space to highlight further accessibility needs. Here we outline practical considerations for involving deafblind people in research conversations.

### Meeting room layout and space requirements

The layout of meeting space is important for setting the tone and expectation for a meeting. A boardroom style layout (where seating is often around a single, long table, sometimes with a seat at the head of the table) often conveys a sense of formality and business-like activity and allows for more people in a smaller space. In contrast, clusters of tables can set expectations towards a more collaborative, discussion-based way of working but a larger and more flexible meeting room is necessary.

For this discussion group, the research team choose a boardroom style layout that would allow for the accommodation of a larger number of people; eight contributors, three interpreters; three researchers and the facilitator. Through the facilitation process we encouraged humour, freedom for contributors to speak without judgement and did not overly emphasise ground rules and rigid adherence to a tight agenda, to ‘break down’ any subsequent sense of formality in the room.

A technique often used in the facilitation of involvement activities is to intersperse researchers among contributors as a way of helping to break down ‘hierarchy’ and remove the sense of ‘them and us’ from the room. In this instance, the facilitator and research team were at one end of the table to create a single focus. Having the facilitation and research content in the room directed from a single location meant contributors did not need to continually reorient themselves, which can be challenging with a limited visual and hearing range.

**Fig. 1:**
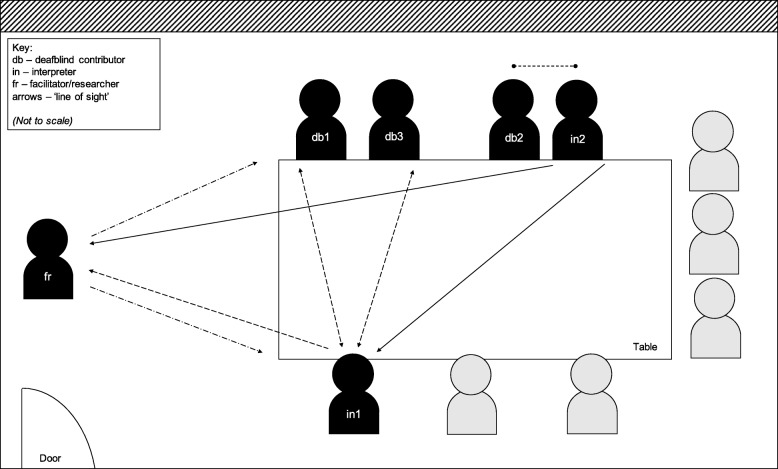
Discussion group layout, showing the position of deafblind individuals relative to interpreters, facilitator and researchers and other contributors. The facilitator and researchers [fr] took up a position at one end of the room, but not at the table, and spoke towards attendees (dash-dot arrows). Deafblind individuals requiring sign language interpretation [db1, db2, db3] sat with their backs to the window (diagonal hatching). For BSL interpreting, interpreters [in1] took up a position closest to fr, db1 and db3 that gave them the best lines of sight (dashed arrows). For manual/hands-on signing (double arrow with round ends), interpreter [in2] and db2 sat further back in the room giving in2 sight of both fr and in1 (solid arrows). In2 also sat slightly behind db2 to allow the best access for on-body signing as required

The research team observed that this layout also enabled contributors and interpreters to adopt a comfortable distance from one another, and the researchers, to give clear lines of sight for hand and facial cues to facilitate simultaneous translation of discussions (see Fig. 1). Working with interpreters and space requirements is further discussed under *Sign Language and Interpretation*.

When working with visually impaired audiences’ space is vital. Some contributors required a white cane, human guide or guide dog to help them with navigation and the research team did observe some contributor difficulty in navigating this narrow space. Therefore, it is important to reach out to contributors early on and ask about space and navigation needs to aid in finding somewhere suitably sized. Be aware that individuals who make use of additional support to assist in their navigation may not think to volunteer this information as it is merely part of their everyday routine. It should be noted that guide dogs need space to lie down, preferably near their owner, and when holding extended meetings, guide dogs also need refreshment, and a break to accommodate a walk.

### Sign language and interpretation

Sign language is a term that is poorly understood. There are many forms of sign language with most countries having their own version. Even between countries that share a common spoken or written dialect (e.g. English), forms of sign language may not be interchangeable (e.g. BSL and American Sign Language) [3].

Sign languages are also not typically a word-for-word, ‘physical’ translation of a spoken sentence. Sign languages have their own grammar, syntax and language rules, and as with spoken languages, have evolved and changed over time. For deafblind individuals, there are further adaptations of sign language whereby the interpreter is directly signing onto the body of the deafblind person. Table 1 provides a list of the forms of sign language and other forms of interpretation commonly used by deafblind individuals in the UK.

**Table 1:** List of the common types of interpreting used by British deafblind individuals

Forms of sign language interpretation	Definitions
British Sign Language (BSL)	The preferred sign language of the UK, with a vocabulary, grammar and syntax which is different from spoken and written English. Most BSL/English interpreters work simultaneously. Simultaneous interpreting involves interpreting in ‘real time’. BSL uses a mixture of gestures and hand movements, body language and facial expressions to facilitate a two-way exchange between the interpreter and the person with hearing impairment. The interpreter translates spoken words and signed language in two directions. In addition to interpreting, the interpreter must also act as a bridge between individuals, relaying tone, intentions and emotions.
Adaptions of BSL used by deafblind individuals	
Deafblind manual	An adapted form of fingerspelling taken from BSL. Each letter is spelt out on the hand, enabling communication by touch alone
Hands-on	Signing is performed directly on the hands of the deafblind person, so they can feel the signs being used
Social haptic	Information about what is happening in the surrounding environment, such as the mood and the activities taking place, are signed directly onto the deafblind person’s body
Visual frame	When a person’s field of vision is severely restricted signing can be conducted in a smaller signing space to fit within the field of view
Other forms of interpretation used by deafblind individuals	
Lipspeakers	A hearing person who has been professionally trained to be easy to lipread. Lipspeakers reproduce clearly the shapes of the words and the natural rhythm and stress used by the speaker
Makaton	More commonly used by people who have learning difficulties. Rather than a language, Makaton is a communication method used to portray simple instructions or feelings as opposed to conversation or concepts
Sign Supported English (SSE)	SSE uses a mixture of lip patterns and the signs from BSL but in the order that the words would be spoken in English. SSE is increasing in use, reflecting the increased support for hearing loss in mainstream schools
Speech to text (palantypist)	A speech-to-text reporter (STTR), also known as a captioner or palantypist, is a person who listens to what is being said and inputs it, word for word, using an electronic shorthand keyboard or speech recognition software. It allows the spoken words to be typed on a screen and read, it is akin to live subtitling

The costs associated with sign language interpreters are one area that can be a barrier to including deafblind individuals in conversations. As with any language, interpretation costs can mount up depending on the number of times and length of time working with the interpreter. Therefore, such costs may become prohibitive both within the research budget as well as for any involvement activities, which have limited funding sources. However, under the 2010 UK Equality Act people with deafblindness should expect an interpreter/lipspeaker to be made available [21]. Working with someone who has both sight and hearing impairment requires specific skills and familiarity. There is also expertise needed for signing within a research and health context [3, 4]. During this discussion group, we were fortunate that contributors were able to bring their own experienced interpreters.

With such a range of different sign language needs, providers may not always be able to offer appropriate interpreters that meet deafblind people’s requirements. Individuals may already have access to interpreter support either through a friend or family member or if they receive allocated hours of support with a personal budget through programmes such as the UK Government’s Access to Work and reasonable adjustments policies [22, 23]. Partnering with hearing loss and sensory disability charities may be another route to access qualified interpreters. Within the school and hospital setting, sign language costs may be covered by existing language interpreter budgets rather than as part of an individual’s disability provision.

Sign language interpreters are qualified translators and, unless explicitly requested by the person requiring interpretation (e.g. a preference for a friend or family member), any interpreters should be registered with the *National Registers of Communication Professionals working with Deaf and Deafblind People* (NRCPD), a voluntary organisation who regulate communication and language professionals who support deaf and deafblind individuals [24].

When working with interpreters there is a need to communicate as far as possible in plain English, as jargon and complicated scientific terms cannot be translated easily and rely on fingerspelling. Limiting the need for this where possible is helpful [4]. For this discussion group, the research team were also asked by a contributor to provide outlines of our presentations in advance to help interpreters prepare to sign on the day.

During the development of this paper, contributors also raised the need for quiet spaces for interpreters to hear what is said, translate accurately and accommodate the limited hearing range of deafblind people. There also needs to be good lighting with no obstructed view of, or windows behind, the speaker or interpreters for individuals to view signing as clearly as possible (Fig. 1):

*“The location of the meeting room is also more critical for deafblind [people]; considering lighting and background noise levels, which all impact the ability to participate fully without distractions.” (C1)*

If you are working with manual signers, they will need the room layout to be able to accommodate them sitting side-by-side or behind the deaf person. Before this discussion group, we were not aware that a manual signing interpreter would be attending on the day. While the research team were not able to provide a pre-briefing for this interpreter, the interpreter said that being able to observe the BSL signing during discussions helped them with translation for the manual signing user (Fig. 1).

Reading sign language can be physically and mentally exhausting, so it is important to give people an opportunity to take regular breaks:

*“Concentration when you do not hear and rely on hearing aids or sign language is more difficult than for those with ‘normal’ hearing which means the [deafblind person] will get tired more quickly, the requirement to have regular breaks to relax for a few minutes will really help.” (C1)*

Signing is also exhausting for interpreters. Having several interpreters who can swap over and offering shorter sessions with regular breaks, will help this.

Not everyone who is deafblind uses sign language, particularly those who lost their sight and hearing later in life. There are additional options available including lipspeakers, who are trained to be easy to lipread. There are also speech to text captioners (otherwise known as palantypists) who can subtitle a meeting live in the room, so the meeting space may need to include appropriate audio-visual facilities. Many of the same considerations such as costs and NRCPD registration apply to these other interpreting services as well.

### Other communication support needs

There is a growing role for assistive or inclusive technologies to enable deafblind people to communicate with broader audiences. Traditionally, Braille was a primary communication method for deafblind people alongside sign-language [25]. However, Braille users now only account for around 1–2% of deafblind people in the UK [25], as it is difficult to learn for those who lose sight later in life or less necessary for those who retain sufficient vision in order to read (with or without the use of magnification or similar visual aids) [26, 27]. While there remains a need to consider the use of Braille devices, which may have specific computer platform and power source requirements, this is unlikely to be a priority consideration for most deafblind people [25].

In many instances the sight and hearing loss needs of individuals with deafblindness may be compensated for by technologies such as digital hearing aids and magnification devices. In many scenarios, audio or video recording of a meeting (with informed consent) and providing minutes or a detailed transcript afterwards might be all someone with a milder hearing or visual impairment requires. There are additional options however for those needing more support in the room to be involved in the conversation (Table 2).

**Table 2:** List of communication support and technologies which can improve information accessibility for deafblind people

Tools and support	Definitions
Audio Induction Loop	A hearing loop (sometimes called an audio induction loop) is a type of sound system for use by people with hearing aids. It is a magnetic system using wireless signals that allows the speaker’s voice to be transmitted by microphone directly to the hearing aids/cochlear implant when using the ‘T’ (Telecoil) setting.
Electronic information	Electronic sources of information need to be in editable formats (i.e. not PDF), so the reader can change the font/colour and size to suit their needs and be written in screen reader-compatible formats (e.g. use of navigable headings and pictures with alt text). Information on websites should be accessible to braille readers and have a BSL version with subtitles.
Printed documents	Large print resources available in easy read formats including high contrast (e.g. black text on a yellow background), large print (at least 18 point) sans-serif fonts (e.g. Arial), suitable pictures used (if applicable) to illustrate meaning.
Recorders and notetakers	A video or audio recorder can be used to capture the content of the meeting or a notetaker can provide written or electronic minutes of a meeting.

Many meeting facilities will now offer an inbuilt audio loop facility. Portable systems can also be purchased for a few hundred pounds and are a simple solution for those who use a hearing aid. From the research teams experience of working with visually impaired audiences, the need for printed documents or slide presentations in a meeting varies. Depending on the visual range of the audience, it may be preferable not to use written or visual materials unless they are necessary to help convey understanding of a particularly complicated concept. Where printed documentation is required, providing these in editable electronic formats in advance of the meeting will allow contributors to become familiar and to put them into formats to meet their accessibility needs such as high contrast and large, visually accessible fonts.

It is increasingly common for those with sight and hearing impairment to rely on smartphones, tablets, e-readers and laptops in day-to-day life and for meetings.

There is now a broad range of devices on the market, available at a range of prices, which provide access to an array of accessibility features to magnify, reformat or to enable text to speech or speech to text translation; either inbuilt or through an increasing number of free and pay-for applications [26–28]. Therefore, the need for power points and access to wifi facilities should also be a consideration.

There is investment being made in ‘wearable’ assistive technologies. For signing there have been developments in sensory gloves, which contain sensors that detect the letter being signed and relays it to a screen or voice synthesiser to translate. There are also hand-tapping devices, where an operator can input what is being said on a keyboard, which is then tapped out on the hand of the deafblind person [25]. For visual impairment, there are head-mounted technologies that include smart glasses; glasses mounted cameras that can read text and recite it back and headsets which can magnify and receive live feeds from television and monitors [28]. These technologies continue to advance as developers seek to improve their accuracy and accessibility but some require the user to receive training. While there are a number of charity, educational and government funding schemes available to increase access to such devices, not everyone is eligible and the prices of such devices remain high, which keeps them currently out of mainstream use [25, 26, 28].

### Emotional support and wellbeing

People who have sight and hearing loss like USH can experience low mood, anxiety and feelings of isolation [3, 4, 8], as do those with other rare diseases [29, 30]. It is therefore important to appropriately manage people’s expectations of research, particularly when discussing the possibility of treatment.

When designing prospective research studies, the stage and progression of the condition or those most frequently seen in clinic can influence the decision on research direction and what to prioritise. During this discussion group, conversations about not all subtypes of USH benefiting from any given research project, raised the important notion of ‘disappointment’ in those who wouldn’t benefit:

*“For me, it was the clarification that you were [not including my subtype] first. My initial response was disappointment.” (C3)*

For individuals who go on to become participants in research studies, months if not years of having to fit one’s personal life around schedules of follow-up appointments lies ahead. Additional stress may arise should interventions be proven not to be tolerable or show no benefits. Worse, interventions could prove to be beneficial but be withdrawn at the end of the study period because of the absence of a license. Contributors showed concern over the potential for further anxiety or stress caused by such a scenario:

*“I guess if you were lucky enough to take part in the trial and you did the year, then you’ve got a year without the drug, but then a year plus before NICE approves it, and you’ve seen positive signs, you’re thinking god I wish I could still be taking this.” (C1)*

Contributors to these discussions advocated for future studies in USH to provide the opportunity to refer for counselling and, as part of the patient information, clear guidance on accessing patient organisations. The benefits of peer-to-peer support for people living with conditions like USH were highlighted as being important not only for daily living, but to potentially benefit those participating in research, especially if that support was between people also on the study:

*“I wondered as well as the genetic counsellor support, whether there could be peer-support as well with those on the trial, that we can keep one another motivated. I just think that could be a useful tool as well.” (C2)*

The potential impact on emotional wellbeing whilst taking part in research also raised questions of whether an assessment of mental health such as potential for low mood and anxiety should form part of standard study enrolment procedure:

*“There are so many people with Ushers who are really, really low. I would worry for them to go on [treatment for] that year and really get something from it and then for them to have a year not getting [any treatment]. [Is] a psychological test needed at the beginning?” (C2)*

Conversations about emotional wellbeing concluded that for those with USH it was vital that studies made access to wrap around counselling and peer support both easy to access and embedded in the research from start to finish:

*“I think it is important that people know there is support available before, during and after. That’s important in terms of providing information but being realistic for people as well” (C3)*

There is some evidence that for those with rare diseases, the family may have the greatest expectations for the research [30]. An awareness of the emotional impact of research for relatives was apparent in discussions with contributors who felt there was a need to manage the expectations of family members, not just for their own emotional wellbeing but for that of the study participant as well:

*“My mum, she’s very, very hopeful. If we attend [the hospital] she’ll draw the most positive aspects of any discussion we may have. Some form of information for the family as well, where expectations for parents, relatives may be too much and would make it more difficult for those individuals in the trial.” (C4)*

Emotional wellbeing was also not just a concern in research participation but also within involvement activities, where sensitivity to people’s health journey is paramount [31]. However, when facilitating a conversation with people who are deafblind, sensitivity in how that dialogue unfolds and the interactions that surround that are equally as important:

*“A key consideration is to have an understanding and considerate chair, or facilitator, for the focus group. Deafblind [people] in a group will all have different levels of sight and hearing, some will use sign language and interpreters, which means that the speed of the session needs to accommodate all the patients in the group to ensure their understanding of the topic is clear and their views are heard.” (C1)*

### Checklist

It has been our experience that while researchers working in the sight loss and vision sector are aware of the existence of recommendations outlining the accessibility needs of people with visual (and hearing) impairment [1, 2, 15, 16, 25–28], as well as deafblind awareness training [31], uptake is low. Patient-facing materials regularly do not meet the needs of the end-user and a one size fits all approach is often taken towards accessibility and support needs [6]. The ‘workaround’ for this is often to ask individuals to have someone with better vision read materials to them, rather than creating the right formats at the start to enable independent decision making.

Discussions with contributors at this involvement activity identified four areas of support and accessibility to consider to facilitate a conversation about research with deafblind individuals, whether that be one of involvement or discussion with an individual about their enrolment as a research participant:

- use appropriately sized, flexible meeting space with good lighting control and sound proofing
- identify the right form of interpreter support for individuals
- ensure availability of appropriate print formats and the ability to support any requirements for electronic communication tools
- manage expectations of research and make any necessary emotional support available to individuals and their family members at all stages of research

We have developed a checklist to help guide the planning for a face-to-face discussion about research with people who are deafblind (Table 3). The checklist has been informed by the themes arising from our own discussion group and supplemented by broader accessibility recommendations. With this checklist we have consolidated and streamlined the considerations for working with deafblind people to make them less daunting to implement. We have tried to cover a depth and breadth of accessibility needs and to reinforce the need for earlier interactions with contributors. These points reflect feedback from our own conversations on the importance of including a range of voices in the discussion that are representative of the broad spectrum of deafblindness:

*“It is essential to get a mix of [deafblind people] with different levels of disability to ensure all views are considered” (C1).*

**Table 3:** Checklist of recommendations for the inclusion of people with deafblindness in a discussion group

Considerations	Recommendations	Check
Room	Large, quiet space with a flexible layout (i.e. not fixed tables and seating) and can accommodate more people than anticipated and with access to an outside area	
Lighting	Room provides control over levels of light, both natural (e.g. windows have blinds) and artificial (e.g. dimmer switch or the ability turn off groups of lights around the room) sources	
Agenda	Agenda is broken down into 20 to 30-min sessions with 10 to 20-min breaks in between	
Documents	Printed materials are in large print (18 point and above), sans-serif fonts (e.g. Arial) and high contrast (e.g. black text on a yellow background) or in braille formats as required	
	Documents have been sent out in advance; either printed or in a screen reader / text-to-speech friendly format (e.g. not PDF)	
Interpreting/lipspeaking	Attendees have confirmed if they will be providing their own interpreters; if so, attendee/interpreters have confirmed any requirements for interpretation	
	If you are required to provide interpreters:	
	Interpreting needs of attendees have been confirmed (e.g. British Sign Language, hands-on signing, lipspeaking etc.)	
	At least two interpreters (to allow interpreters to alternate and to have a break) have been identified, and both are NRCPD registered	
	Interpreters have experience working with individuals who are deafblind	
	Interpreters have experience working with scientific content (e.g. health, biomedical research etc.)	
	Relevant materials relating to the content of the meeting, have been sent in advance to interpreters	
Audio communication tools	Room is fitted with an audio induction loop	
	Speech to text (e.g. captioner, palantypist) is available for those who cannot use the available sign language options	
	There is sufficient easy access to power points for people’s accessibility aids (e.g. smartphones, tablets, e-readers, laptops etc.)	
Emotional wellbeing	Relevant information on/access to patient organisations, peer-to-peer support; counselling is available	
	Skilled/trained and sensitive facilitator	

## Discussion

The opportunity to include deafblind people in conversations about research, while providing significant benefits to both the research team and contributors [17–20], is not without its challenges. Our aim in writing this paper was to consolidate both our experience and input from people with USH, to inform the most important considerations.

There are already a number of documents and websites in the public domain providing recommendations on the accessibility needs of people who have sight loss [15, 16, 28], hearing loss or both [1, 2, 25–28]. Much of the guidance and support we have identified here comes from deafblind and sensory disability charities [1, 2, 15, 16, 28]. This information comes in the form of guidance documents as well as personal recommendations about accessible technologies. Some charities also offer training courses and consultancy in how to work with deafblind individuals and increase accessibility to improve the experience for all [28, 32]. However, what we learned from our review of the literature is that these resources can be difficult to find if you do not know where to look in the first instance and tend to be lengthy and broad in their coverage of the available accessibility support. From this involvement activity it is also clear that there is no standardised method of communication [6], which may make it difficult to know where to start.

Reporting of research and sharing experiences of the best methodologies in involvement is limited. There are particular gaps in information relating to overcoming barriers and working with people who are identified as having a disability or a mental health illness (including low mood, anxiety and depression) [17, 19], and therefore, by extension, people with a rare disease [29, 30].

A similar information gap also exists in clinical research participation. The PREDICT study, while taking a first step in identifying that the exclusion of people with sight and hearing impairment from research is ‘poorly justified’, have not gone as far as to define any specifics to address this [9]. Of the four registered clinical trials involving participants with USH we found during our literature review [13, 14], we have not found any published evidence relating to how these studies have targeted their communication approaches to meet the needs of people who are deafblind. As the studies close and are written up, we hope to see that these approaches are included in the methodology with sufficient detail in order to allow other researchers to facilitate appropriate communication support for people who are deafblind.

The aim of this paper and our checklist (Table 3) was not to provide a detailed singular experience of working with people who are deafblind but provide guidance that is more broadly applicable. This is why the checklist is framed to encourage people to speak to their deafblind contributors early on about what their individuals needs are.

Within the checklist we have distilled the guidance from existing resources namely around document development, interpreting/lipspeaking and audio/visual communication tools into to those points which our deafblind contributors felt were most important [1, 2, 6, 25–28]. With the checklist we want people to take into consideration that for a conversation to be inclusive with representative perspectives, you must be able to accommodate the wide variety of accessibility and communication needs people have. We are also seeking to be mindful of the differences in people’s local resources as a limiting factor and potential barrier to the inclusion of deafblind people in research conversations [17, 19].

In this paper we have also accounted our experience of the practicalities of the meeting space and agenda when holding a discussion with people who are deafblind, which is not mentioned in the literature. The location, flexibility and facilities of the meeting room are critical for deafblind individuals. Lighting and background noise affect the ability of people with deafblindness to concentrate and participate. Relying on hearing aids or reading sign language is difficult and both the deafblind person and their interpreter can tire quickly. The requirement to have regular breaks and to relax for a few minutes helps. Space is also important to consider, both to enable the interpreter and deafblind individual to arrange themselves optimally but also to ensure that trip hazards and narrow spaces do not impede a deafblind person’s ability to move using their principle method of navigation [6]. Contributors may also rely on a range of written and technology formats to help with discussions, so any meeting space must accommodate for these [1, 2, 6, 25–28]. Creating a space that shows consideration of the meeting’s purpose and one’s audience serves everyone.

Finally, in the checklist we have also advised researchers to consider people’s emotional wellbeing. This is another area we have not seen mentioned in existing accessibility recommendations for working with people sight and hearing loss. Conversations about research, particularly when discussions are about potential treatments and cures for life impacting conditions, must be handled with compassion and consideration [19, 29–31]. The value of support in the form of information and counselling, including from peers, has previously been reported as being important for people with sight and hearing loss and for those with rare disease, but is all too often not made readily available [3–8, 29, 30]. For our deafblind contributors emotional support for them and their families was equally as important as accessibility requirements. Our contributors highlighted such support as being necessary at all stages of the research cycle, and to manage the expectations of relatives, which could be a source of additional emotional burden. The role of the facilitator in discussions was also identified as being important to make the overall experience one where people felt they can contribute openly without being under time pressure and that their views are of value to support the research process. Education and training in facilitation for professionals has been identified previously as being important for effective involvement activities [19].

The authors recognise that the recommendations made here are from a UK perspective and have had the benefit of input from one of the UK’s major sensory impairment charities and deafblind people directly [1, 2, 21–23, 27]. There will perhaps be barriers to fully realising the recommendations in this checklist that the authors have not come up against, such as access to people with deafblindness. A known challenge, particularly in non-clinical research, is that researchers may have never met someone with deafblindness or have relationships with medical institutions, charities or organisations who could connect them with deafblind people. Therefore, there is a greater emphasis on forward planning in order to make these connections.

When you have made a connection with someone who is deafblind their degree of sight and hearing loss will also directly influence your ability to communicate with them. With more severe impairments you may be limited in the types of communication possible. For some people, text-based communication like email or text message might be their preferred method of communication. Some people may be able to use the telephone or even video conferencing but may need an interpreter or assistive technology support, so forward planning is again important [1, 2, 27].

The availability of resources for deafblind individuals both across the UK and aboard may differ from what we have suggested here and for some, limited local budgets may make some of these recommendations challenging to implement. For our involvement activity, which was the basis of this paper, access to local core resources (such as meeting rooms) and the generosity of contributors in providing their own interpretation and accessibility support meant our cost impact was minimal. Budget limitations are of course a concern. People who are deafblind may find public transport difficult to use and so private transport costs may also be a factor in planning [3, 6]. Your own institution may not have suitable meeting facilities, which could result in additional venue hire costs. A group meeting, which is the premise of this checklist, may not be suitable for everyone. Some people may be more comfortable to meet you in their own home, which is more accessible for them, but may incur travel costs [19]. Therefore, when developing budget plans, early conversations with contributors about accessibility needs as well as with local research offices and finance and procurement teams are essential. Hopefully, in most organisations there will be preferred supplier agreements and special contractor rates in place that can pass on cost savings. Sensory disability charities may also be able to advise. It is also worth bearing in mind that though it is not a cost directly relating to accessibility, it is considered best practice where possible to remunerate people for their time and expenses to be involved [19].

While our recommendations come from working with people with the most prevalent form of deafblindness worldwide, due to the multicultural nature of society and the spectrum of sight loss and hearing range across the deafblind community, there may be additional needs not covered here.

## Conclusions

Here we have documented an account of the inclusion of deafblind people in a conversation about research. We highlight that people rely on a range of different approaches for their individual communication and accessibility needs as well as the importance of finding the right physical space to hold such conversations. Contributors also strongly advocate for counselling and peer support for themselves and their relatives as part of an enablement strategy. By supplementing this real-world experience with input from deafblind people, the sensory impairment charity Sense and other sources; we have developed a checklist of flexible but practical recommendations to support others in holding similar conversations. However, barriers still remain. Working with specialists like interpreters and palantypists or the need for private car hire or use of a suitable external meeting venue may impact on local budgets. Therefore, early planning is important to identify what access to resources deafblind contributors may already have, determine what may already exist within your organisation and to include suitable allocations within future funding bids.

## Abbreviations

AS: Andi Skilton
BSL: British Sign Language
C1–4: Contributors
EB: Emma Boswell
ICON-1: Improve Cardiovascular Outcomes in high-risk patieNts with acute coronary syndrome
KP: Kevin Prince
MM: Mariya Moosajee
NCRCPD: National Registers of Communication Professionals working with Deaf and Deafblind People
PDF: Portable Document Format
PF: Priya Francome-Wood
PPI: Patient and Public Involvement
PREDICT: PaRticipation of the ElDerly In Clinical Trials
SSE: Sign Supported English
STTR: Speech to text Reporter
USH: Usher syndrome(s)
